# Formation and evolution of idiopathic lamellar macular hole-a pilot study

**DOI:** 10.1186/s12886-022-02669-4

**Published:** 2022-11-14

**Authors:** Cheng-Yung Lee, Yun Hsia, Chung-May Yang

**Affiliations:** 1 Department of Ophthalmology, National Taiwan University Hospital, No.7, Chung Shan S. Rd. (Zhongshan S. Rd.) , Zhongzheng Dist., Taipei, 100225 Taiwan (R.O.C.); 2 Department of Ophthalmology, National Taiwan University Hospital Biomedical Park Branch, No. 2, Sec. 1, Shengyi Rd., Zhubei City, Hsinchu County 302 Taiwan (R.O.C.); 3 Department of Ophthalmology, College of Medicine, National Taiwan University, No. 1, Sec. 4, Roosevelt Rd., Taipei, 10617 Taiwan (R.O.C.)

**Keywords:** Lamellar macular hole, Developmental process, Degenerative lamellar macular hole, Epiretinal proliferation, Optical coherence tomography, Visual acuity

## Abstract

**Background:**

The developmental pathways and subsequent evolutional processes of idiopathic lamellar macular hole (LMH) were studied with spectrum domain optical coherence tomography (SD-OCT).

**Methods:**

Twenty-seven eyes of 26 patients of idiopathic LMH with pre-LMH SD-OCT available were retrospectively reviewed. Relevant OCT parameters and best-corrected visual acuity (BCVA) were collected and analyzed.

**Results:**

Four types of developmental pathways of idiopathic LMH were noted. Type 1 (5 cases), involved disruption of a foveal cyst from vitreomacular traction. Type 2 (10 cases), demonstrated rupture of parafoveal cysts or schisis mainly from epiretinal membrane (ERM). In type 3 pathway (5 cases), a central intraretinal cyst formed under tight ERM with subsequent cyst roof dehiscence. Type 4 (7 cases), showed gradual loss of foveal tissue without cystic lesions from ERM traction. There was no statistically significant change in BCVA during LMH formations or subsequent evolutional processes in any types of the developmental pathways. Three cases developed epiretinal proliferation (EP) during evolution, which showed tendency of decrease in BCVA. Among the three cases, one later developed the degenerative configuration.

**Conclusions:**

In summary, four types of tractional developmental pathways of idiopathic LMH were identified. BCVA was relatively stable during LMH formation and follow-up. Deterioration of visual acuity were found in cases that developed EP during evolution. Transformation into degenerative configuration might be possible after LMH formation.

## Background

Lamellar macular hole (LMH) is a specific type of vitreo-macular disorder characterized by a defect in the inner retinal layer [1]. It may come from rupture of the inner retinal cysts or from epiretinal traction [2, 3]. The advances in optical coherence tomography (OCT) in recent years have facilitated our understanding of the detailed structural changes and the evolution of LMH.

Recently, Govetto et al. proposed that idiopathic lamellar macular holes might develop via either tractional or degenerative pathways [4]. However, whether the tractional or degenerative types of idiopathic lamellar macular hole represent two distinctive formation pathways or just different stages of the disease evolution is under debate [1, 4]. More recently, Hubschman et al. published an expert consensus on the OCT-based definition of LMH [5]. The authors proposed that LMH-related lesions can be separated into three subgroups. However, the developmental pathways of each subgroup remain unclear, and whether there are different formation pathways of idiopathic LMH have not been clearly elucidated.

In this study, we investigated the developmental pathways of idiopathic LMH and observed the evolution after its formation through sequential OCT images in order to better understand the structural changes of this specific entity.

## Methods

This was a retrospective, observational, chart review study. All cases diagnosed as idiopathic LMH according to OCT morphologic criteria [6] by the ophthalmology department in one tertiary hospital between January 2007 and May 2020 were reviewed. Of these, only those cases with OCT records prior to the development of LMH, with sequential OCT records after LMH formation, and without prior surgical interventions were included in the study. Cases with other clinically significant retinal diseases, such as vascular occlusion, severe non-proliferative or proliferative diabetic retinopathy, high myopia with axial length > 26.5 mm, history of major ocular trauma, or ocular inflammation diseases, were excluded.

Follow-up interval after LMH formation is defined as the time interval between the date of the first OCT examination with the presence of LMH to that of the latest OCT examinations. Patients with follow-up after LMH formation shorter than 6 months was excluded.

Serial OCT images, as well as medical records for each case, were collected and analyzed. This study was conducted according to the principles of the Declaration of Helsinki. The study was also approved by the Ethics Committee and Institutional Review Board of National Taiwan University Hospital.

Medical records of all the cases, including best-corrected visual acuity (BCVA), axial length, ocular fundus changes, and basic demographic information, were collected. All the OCT examinations were conducted with the following machines: Optovue Avanti™ OCT (Optovue, Inc., Freemont, CA), Optovue RTVue XR™ OCT (Optovue, Inc., Freemont, CA), or Optovue RTVue™ OCT (Optovue, Inc., Freemont, CA). In all the examinations, standard 6 mm or 9 mm OCT images in the macula were obtained. In short, six evenly distributed radial scans centered at the fovea were performed; horizontal and vertical raster scans were also performed over the macular area to obtain detailed images of LMH and macular structures. On the OCT images, several parameters were manually measured using the software calipers, including the diameters of the outer and inner layers of LMH and the thinnest foveal thickness.

The diagnosis of LMH follows the OCT-based criteria proposed by Witkin et. al. [6]: (1) irregular foveal contour; (2) break in the inner fovea; (3) intraretinal split caused by the separation of the inner from the outer foveal retinal layers; and (4) absence of a full-thickness foveal defect. In all cases involved in this study, blue-fundus autofluorescence images (B-FAF) were acquired as confirmation of foveal tissue loss. The definition of macular pseudohole used in this study was consistent with the diagnostic criteria established by Hubschman et al. [5]: (1) epiretinal membrane (ERM) sparing the foveal area; (2) thickening of the retina at the parafoveal area; and (3) verticalized or steepened foveal structure. Furthermore, cases defined as macular pseudohole should not have hyperfluorescent signal in B-FAF. [7, 8] To fulfill the definition of degenerative lamellar macular hole (DLH), an LMH must meet at least 3 of 5 diagnostic criteria proposed by Govetto et al. [4], which are: (1) an inner-on-outer diameter ratio shown to be bigger than ½; (2) ellipsoid zone disruption; (3) round edge cavitation; (4) foveal bump; and finally (5) the presence of epiretinal proliferation (EP). The diagnosis of each included patient was checked by two retinal specialists at National Taiwan University Hospital. If opinions differed, discussions were held until consensus was reached.

For each group, age, OCT images, BCVA before and after the development of LMH were collected. In addition, the BCVA and OCT images during evolutional process of each LMH were also collected. The evolutional BCVA changes between those with EP and without EP were also collected and compared. The presence of tractional or degenerative features in each case was specifically looked for.

## Results

Twenty-seven eyes of 26 patients were included in this study. Their demographics information is displayed in Table 1. Four developmental pathways of idiopathic LMH were identified (Table 2).

**Table 1:** Demographics

Number	n	27
Age	year (std)	60.3 (8.3)
Gender ratio	Male: Female	10:17
VA before LMH formation	Log Mar (std)	0.13(0.16)
VA right After LMH formation	Log Mar (std)	0.15(0.16)
VA of the last follow-up	Log Mar (std)	0.22(0.18)
Follow-up time after LMH formation	months (std)	31.8(20.0)

**Table 2:** Sub-group demographics categorized according to different types of developmental pathways

Type	Number	Age	VA before LMH formation	VA right After LMH formation	VA at the last follow-up	Follow-up time after LMH formation
	n	Year-old (std)	Log MAR (std)	Log MAR (std)	Log MAR (std)	Months (std)
1	5	64.6 (8.4)	0.14 (0.16)	0.12 (0.067)	0.21 (0.14)	38.0 (35.0)
2	10	58.1 (5.3)	0.14 (0.21)	0.10 (0.13)	014 (0.12)	31.5 (17.2)
3	5	60.1 (3.1)	0.16 (0.14)	0.19 (0.15)	0.26 (0.19)	29.8 (7.7)
4	7	61.3 (13.6)	0.11 (0.14)	0.22 (0.22)	0.30 (0.25)	26.9 (19.1)

In **t**ype 1, LMH developed via disruption of the inner roof of an intraretinal cyst. It was seen in 5 cases. Initially the vitreomacular traction (VMT) in the fovea created a cystic space in the Henle fiber layer (HFL)/ outer plexiform layer (OPL), the persistence of the VMT inducing the rupture of the inner wall of the cyst, the LMH was subsequently formed. The average BCVA in log MAR changed from 0.14 (standard deviation (std) = 0.16) before LMH formation to 0.12 (std = 0.067) after LMH formation.

In **t**ype 2, the inner medial wall of the parafoveal cysts or schisis ruptures in the end, and LMH developed. It was seen in 10 cases in this study. In these cases, the traction forces exerted by either VMT or ERM led to localized schisis in the HFL/OPL layer around the foveal or parafoveal area. Parafoveal thickening was firstly noted. In later stage, the gradual breakdown of the medial lining of the cysts/schisis at the level of the HFL/OPL led to LMH. The average BCVA in logMAR changed from 0.14 (std = 0.21) before LMH formation to 0.10 (std = 0.13) after LMH formation.

In type 3, a relatively flat-roofed central cyst formed by a tight ERM; subsequent dehiscence of the cyst roof led to LMH. It was seen in 5 cases. Foveal elevation of the inner retina, including nerve fiber layer (NFL) to OPL, or loss of foveal depression is caused by a taut and flat ERM at first. Usually, a single cystic space emerged in the HFL/OPL layer under persistent ERM traction. Subsequent rupture of ILM, and layers involving Muller cell process at the foveal area created a true LMH. For these cases, the average BCVA in logMAR changed from 0.16 (std = 0.14) before LMH formation to 0.19 (std = 0.15) after LMH formation.

**Fig. 1:**
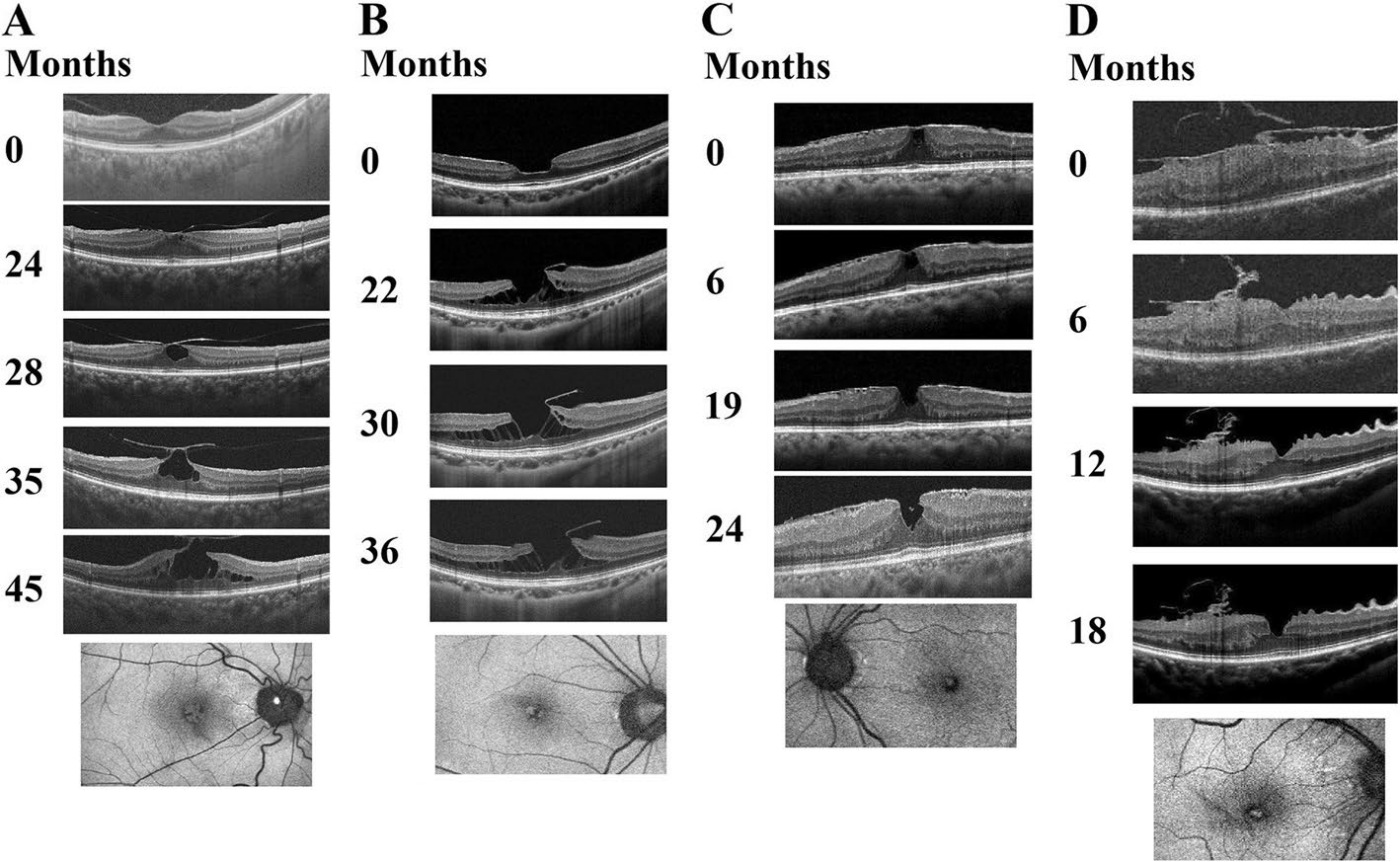
Four types of developmental pathway of LMH. **A** (Type 1): The LMH develops via disruption of the inner lining of an intraretinal cyst from vitreo-foveal traction. **B** (Type 2): The formation of foveoschisis causes the inner medial wall of the fovea to rupture. **C** (Type 3): The rupture of a taut and flat ERM leads to an intraretinal cyst. The rupture of the cyst causes LMH. **D** (Type4): The development of a fovea-sparing ERM in macular area elevates the macula, which subsequently causes tissue loss in fovea and forms the LMH without forming an intraretinal cyst. At the bottom of each column is B-FAF image, showing hyper-auto fluorescence signal, as supportive evidence for LMH

In type 4, LMH developed from gradual foveal thinning. It was seen in 7 cases. The centrifugal traction force provided by ERM initially caused the elevation of the foveal and parafoveal tissue from NFL to OPL. There was no intraretinal cysts typically seen in other developmental pathways. Instead, gradual undermining of HFL/OPL layer extended toward outer retina under the persistent ERM traction led to the LMH, confirmed by FAF. In this type, the average BCVA in logMAR changed from 0.11 (std = 0.14) before LMH formation to 0.22 (std = 0.22) after LMH formation. Figure 1 displayed examples of the four types of developmental pathways.

Among all cases included, 3 cases developed EP during evolutional change. Two of them developed into idiopathic LMH via the Type 2 developmental pathways, and another one developed via the Type 3 pathways (Table 3). The average BCVA in log MAR changed from 0.24 (std = 0.26) right after LMH formation to 0.41 (std = 0.26) at the last follow-up. There was a tendency of VA deterioration in these 3 cases with EP in the end, comparing to those without EP throughout observation period, though the case number was too small for statistical analysis.

**Table 3:** Visual acuity changes between the group with EP and the group not without EP

	Numbers	Age at LH formation	Follow Up Time	VA right After LMH formation	VA at the last follow-up	VA change
	n	year-old (std)	Months (std)	Log MAR (std)	Log MAR (std)	Log MAR (std)
With EP	3	55.7 (3.8)	19.7 (18.6)	0.24(0.26)	0.41 (0.26)	0.16 (0.04)
Without EP	24	60.8 (8.5)	32.6 (20.1)	0.14 (0.15)	0.19 (0.16)	0.071 (0.22)

**Fig. 2:**
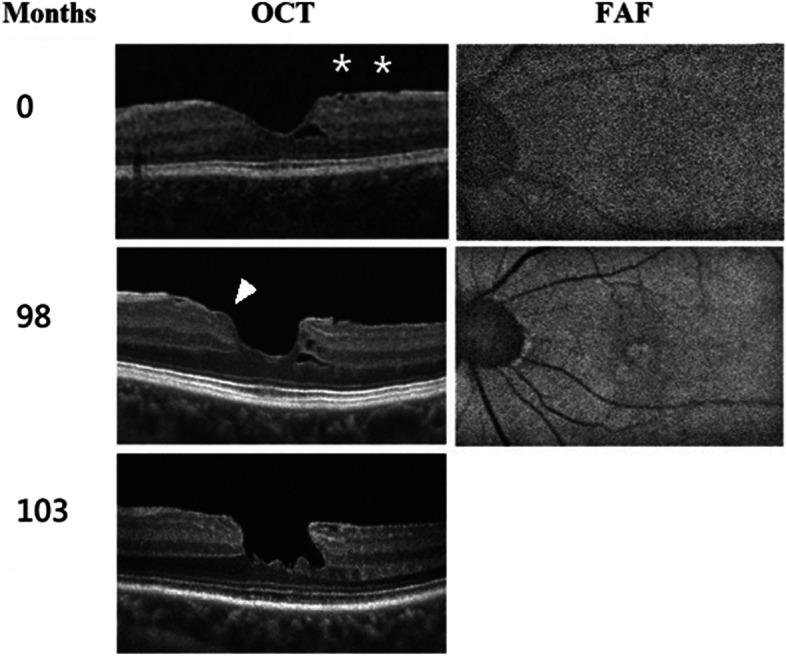
A case of LMH developed via tractional pathway and gradually evolved into degenerative configuration. In this case, LMH developed via type 2 developmental pathway. Initially, ERM, minor internal limiting membrane wrinkling (asterixis, upper right) and a parafoveal cyst were noted (upper left). B-FAF showed no central hyper-autofluorescence (upper right). Widening of the foveal pit and LMH with EP (arrowheads, middle left) developed at around the 8.^th^ year of observation (middle left). At a later stage (lower left), the degenerative configuration emerged, with a round edge and foveal bumps. B-FAF confirmed the diagnosis of LMH (middle right)

In our study, there was no case without EP that progressed into the degenerative type of LMH. Among the 3 cases with EP noted at the last follow-up, there was one case that transform into a DLH (Fig. 2). The formation of the LMH in this case belonged to Type 2 pathway. BCVA right after the formation of LMH was 0.8 on the Snellen chart. After 5 months of observation, EP was noted on the SD-OCT image. BCVA at that time was 0.6 on the Snellen chart. The configuration of LMH fit 3 of the diagnostic criteria for degenerative type LMH, with an inner-on-outer diameter ratio bigger than 1/2, round edge cavitation, and the presence of EP being found. There was no ellipsoid zone disruption.

**Fig. 3:**
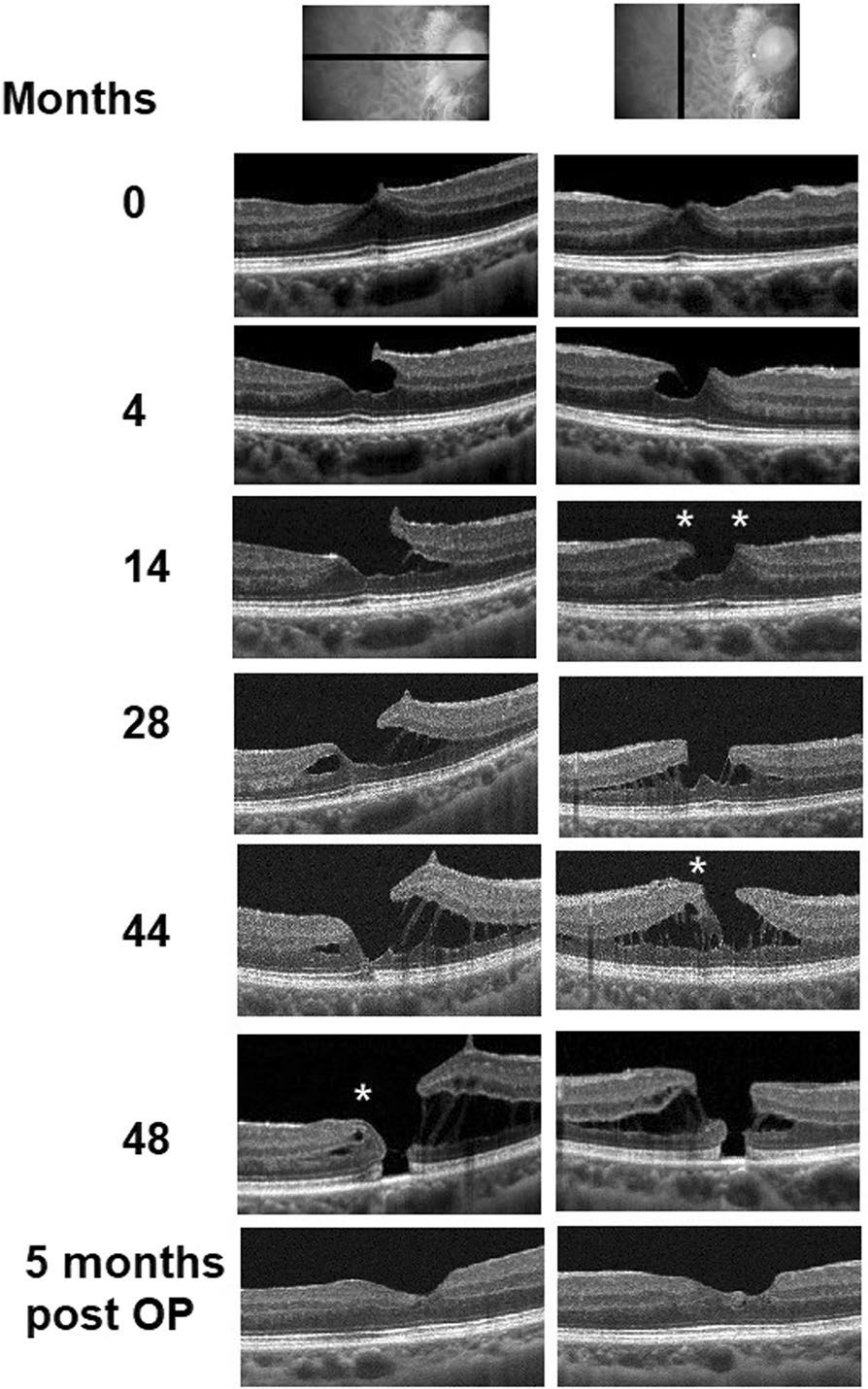
A case of LMH developed into FTMH. This case developed LMH via tractional pathway at around the 4^th^ months of observation. Some EP (asterixis) tissue around the LMH was noted on B-scan image of OCT at the 14^th^ months of observation. LMH progressed to FTMH at the 4^th^ years of observation. Pars plana vitrectomy with inverted internal limiting membrane flap was subsequently performed. the macular hole was sealed 5 months after the operation

There was one case of LMH that later developed into full thickness macular hole (FTMH). BCVA deteriorated as well (Fig. 3). The patient underwent macular surgery, and the macular hole was sealed afterwards.

In addition, there was one patient developed idiopathic LMH via type 4 developmental process. Progressive outer retinal layer disruption, including ellipsoid zone, was noted after LMH formed. The visual acuity deteriorated to 6/20 from 20/20 in Snellen chart. The patient subsequently received vitreoretinal surgery, and the vision was restored.

## Discussion

In this study, by analyzing data in patients with pre- and post-LMH formation, we found 4 distinct tractional developmental pathways of idiopathic LMH. The varied structural changes implied different origins of traction during the formation of idiopathic LMH.

In Type 1 developmental pathway, the intraretinal cyst in the foveal area developed first. The presence of the posterior hyaloid membrane and its point of connection to the inner wall of the intraretinal cyst led to the rupture of that wall, resulting in the formation of idiopathic LMH. This mechanism was regarded as an aborted macular hole in a previous study [9].

In Type 2 developmental pathway, intraretinal cysts or schisis developed in the parafoveal area. The presence of an ERM and the traction generated cause the gradual thinning and the eventual rupture of the medial wall of the parafoveal cysts/schisis, leading to idiopathic LMH.

In Type 3 developmental pathway, the presence of a taut and flat ERM flattens the foveal surface. An intraretinal cyst formed in the fovea. The rupture of the inner cystic wall resulted in a LMH. In this type, the fundus autofluorescence image of the macula was an important tool to differentiate LMH from macular pseudo-hole.

In Type 4 developmental pathway, the presence of an ERM and/or vitreomacular traction caused retinal elevation around the parafoveal area, leaving the foveal area unaffected. In our study, the configuration of this stage showed no tissue loss on the fundus autofluorescence image, and was diagnosed as pseudo-hole. With the persistence of traction, idiopathic LMH subsequently formed. Notably, this type of developmental pathway did not go through intraretinal cyst or schisis stage.

In short, type 1 may represent an aborted stage 1 macular hole process; type 2 derives from paracentral schisis; type 3 results from inner central cyst rupture; type 4 comes from continuous central thinning. The main traction force in type 2 to 4 has been epiretinal membrane. Because the traction forces and directions may be complicated, it was difficult to pinpoint the exact mechanism of LMH formation in each type. The best we can do is to describe and sub-group the structural changes observed around LMH formation as above.

Recently, Govetto et al. proposed that idiopathic LMH could be classified into two distinctive types: tractional and degenerative (4). They hypothesized that the developmental pathway of DLH is distinct from that of tractional LMH, or may be a different reaction to a similar stimulus of the tractional pathway. The high frequency of non-contractile EP observed in degenerative LMH further implies a different developmental pathway. However, the details of that pathway have not been clearly discussed. More recently, Hubschman et al. [5] classified LMH and associated structures into 3 subtypes: LMH; macular pseudohole; and ERM with foveoschisis. In the consensus by Hubschman and other investigators, the diagnosis of LMH requires three mandatory OCT characteristics: (1) irregular foveal contour; (2) foveal cavity with undermined edge; and (3) presence of a loss of foveal tissue. Furthermore, associated pathological changes may include: (1) EP; (2) foveal bump; and (3) ellipsoid line disruption. These diagnostic criteria for LMH were similar to those for the DLH proposed by Govetto et al. [4]. They hypothesized that some LMHs develop from the posterior vitreous detachment and the following partial avulsion of foveal tissue. Whether there are additional pathways causing slow loss of retinal tissue that lead to DLH remain largely unclear.

It has long been thought that tractional forces around the vitreomacular interface play important roles in the development of idiopathic LMH [4, 6, 7, 10–17]. In this study, we defined LMH based on the widely adopted criteria from Witkin et. al. [6]. Under such definition, we ensured that all types of LMH, whether degenerative or tractional, could be recruited. We found all cases of LMH in our study developed via various types of tractional pathways. Tractional development pathways precede alteration of the macular configuration into DLH was found, either from contraction of ERM, vitreomacular traction, or mixed tractional forces around the vitreomacular interface. We were not able to find any LMH developed from the so-called “degenerative developmental pathway.” It is possible that the limited case numbers in our series were insufficient to include all the developmental pathways of LMH. Alternatively, this finding could imply that the “degenerative change” of idiopathic LMH may be a later form of its evolution. We hypothesized that both traction LMH and DLH share a common developmental pathway, in which they are all of tractional etiologies. It might be that different traction directions cause the fovea to go through different evolution processes, with uniformly distributed mild centrifugal tangential traction more likely to induce “degenerative” morphological alterations. Thus, our study findings suggest that instead of being a different entity, the LMH with “degenerative” morphological changes developed as the result of a different evolution from what was essentially a tractional developmental pathway.

**Fig. 4:**
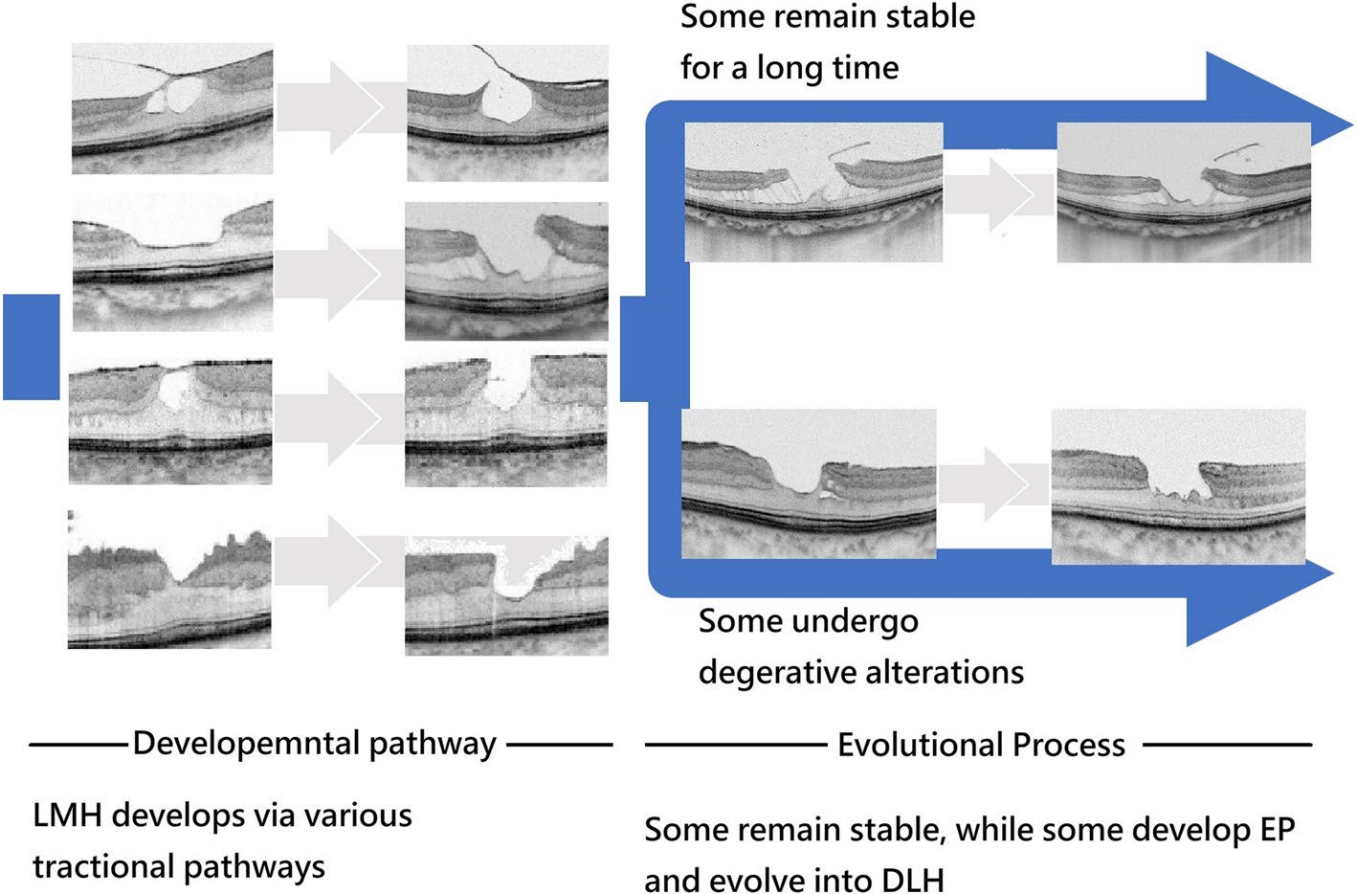
The proposed developmental pathway and evolutional processes of LMH and DLH. In our hypothesis, most of idiopathic LMHs develops via variable tractional pathways. After formation, most LMHs remain stable. On the other hand, EP tissue might develop in some cases. Among these cases with EP, some may evolve into DLH

Compera et al. [18] described the developmental pathway and evolutional process of LMH in a case report that seems compatible with our hypothesis. Recently, Bringmann, A et al. also proposed that the DLH evolved from tractional LMH [19], which is consistent with our findings. In their hypothesis, the DLH, that is characteristic of epiretinal proliferation tissue, is a repairing process derived from tractional LMH, and most LMH developed via traction pathway. Wu et. al. in a recent review article favored the viewpoint that the initial formation step of degenerative LMH comes from tractional event, and epiretinal proliferation degeneration configuration follows [5]. Hsia et. al. in the study of LMH in high myopia also suggested that degenerative configuration appears in the evolutional processes of LMH, and the early developmental process were all tractional [6]. Figure 4 depicts our hypothesis that most idiopathic LMH developed via various kinds of tractional pathways, some remained stable after formation, some, however, developed into DLH.

Although the formation time of secondary LMH have been reported [7, 8], it is difficult to determine the formation time of idiopathic LMH because there is no specific ocular event that can be seen as the starting point of LMH formation. After idiopathic LMH formation, literature review as well as our study showed that LMH may remain structurally and functionally stable throughout long periods [12, 20, 21]; however, some LMH may develop into full-thickness macular holes (FTMH) [12, 22–24]. Among the 27 eyes included in our study, one eye developed FTMH (Fig. 3). Initially, LMH developed via type 2 tractional developmental process. The EP subsequently developed at the edge of LMH, and subtle outer retina disruption was noted on OCT at the 44^th^ month of observation. FTMH developed in the end at the 48^th^ months of observation. Previous studies had shown that for those LMH that eventually developed into FTMH, EP was present in most of the cases [23–26]. Those LMH with the presence of EP is associated with a higher rate of outer retinal disruptions [18, 27–29], which implies weaker outer retinal structure, and thus is more likely to develop into FTMH [23, 25, 28, 30]. Similarly, the higher rate of outer retinal disruption, including ellipsoid zone in such cases also leads to poorer visual acuity, even without FTMH [27, 28].

In this study, we found that there was no significant difference in the changes in BCVA or OCT parameters, including the outer and inner diameters of LMH and central foveal thickness, produced by different evolutional processes. However, for those with EP during evolutional processes, the deterioration in BCVA were tend to be worse regardless of the type of developmental pathway, although the small case number prevented an adequate statistical analysis (Table 3). In short, the type of developmental pathways of LMH does not determine the visual prognosis, since the pathway mainly disrupts inner layers of retina. Instead, other structural changes during evolution, such as the development of EP, the presence of a degenerative foveal contour, or disruption of outer retina layers, dictate the visual deterioration of idiopathic LMH.

B-FAF has been regarded as a helpful tool in differentiating LMH-related lesions [7, 8, 31]. In a previous study, B-FAF showed a clear hyperfluorescent signal around the foveal area even if there was only a small amount of tissue loss [8]. The absence of a B-FAF signal indicated the integrity of the foveal tissue [7, 8]. B-FAF was proven to be a helpful tool in situations where it was difficult to differentiate macular lesions with spectrum domain-OCT images [7]. Consequently, in our study, B-FAF was used as an non-invasive method for assisting the detection of foveal tissue loss.

There were some limitations to this study. Firstly, this is a retrospective study, and it was difficult to collect those LMH cases that also had pre-LMH OCT available. The small sample size may be due to the inherent difficulties to collect the cases with fully documented developmental process of idiopathic LMH, since most patients visited clinic only after they became symptomatic when the LMH already developed. Other developmental pathways might have been found had a larger number of cases been collected. Second, all examinations were not performed at regular interval, so more detailed description might not be possible. However, as far as we know, this is the first study to investigate and propose the developmental pathways of idiopathic LMH through serial OCT images and fundus changes. It may provide background and comparison for future study on the evolution of idiopathic LMH.

## Conclusions

In summary, in this retrospective, chart-reviewing case series, all LMHs collected developed via several types of tractional pathways. Our study suggest that the tractional mechanism dictates the developmental pathways in most cases of idiopathic LMH. While most of LMHs remain stable over long period after formation, some may transform into degenerative configuration in later stage.

## Data Availability

All data generated or analyzed during this study are included in this published article.

## Abbreviations

BCVA: Best-corrected visual acuity
B-FAF: Blue-fundus autofluorescence images
DLH: Degenerative lamellar macular hole
EP: Epiretinal proliferation
ERM: Epiretinal membrane
FTMH: Full thickness macular hole
HFL: Henle Fiber Layer
LMH: Lamellar macular hole
NFL: Nerve fiber layer
OCT: Optical coherence tomography
OPL: Outer plexiform layer
SD-OCT: Spectrum domain optical coherence tomography
VMT: Vitreomacular traction
