# Correction: Formation and evolution of idiopathic lamellar macular hole-a pilot study

**DOI:** 10.1186/s12886-022-02725-z

**Published:** 2022-12-14

**Authors:** Cheng-Yung Lee, Yun Hsia, Chung-May Yang

**Affiliations:** 1 Department of Ophthalmology, National Taiwan University Hospital, No.7, Chung Shan S. Rd. (Zhongshan S. Rd.), Zhongzheng Dist., Taipei, 100225 Taiwan (R.O.C.); 2 Department of Ophthalmology, National Taiwan University Hospital Biomedical Park Branch, No. 2, Sec. 1, Shengyi Rd., Zhubei City, Hsinchu County 302 Taiwan (R.O.C.); 3 Department of Ophthalmology, College of Medicine, National Taiwan University, No. 1, Sec. 4, Roosevelt Rd., Taipei, 10617 Taiwan (R.O.C.)

**Correction: BMC Ophthalmol 22, 432 (2022)**

**https://doi.org/10.1186/s12886-022-02669-4**

Following publication of the original article [1], the author group reported errors in the reference citation.

The corrected text citations are given below:

In page 6, the first paragraph should read, “Thus, initial formation step of degenerative LMH comes from tractional event, and epiretinal proliferation degeneration configuration follows [2] (reference 20 in the original article). Hsia et. al. in the study of LMH in high myopia also suggested that degenerative configuration appears in the evolutional processes of LMH, and the early developmental process were all tractional [3] (reference 21 in the original article).”

In page 6, the second paragraph should read, “Although the formation time of secondary LMH have been reported [4, 5] (references 22, 23 in the original article), it is difficult to determine the formation time of idiopathic LMH because there is no specific ocular event that can be seen as the starting point of LMH formation.”

The original article [1] and the reference list have been corrected”.
